# Residual Stress-Based Soft Robot with Capability for Grasping and Buoyancy Control

**DOI:** 10.3390/biomimetics11050317

**Published:** 2026-05-02

**Authors:** Minchae Kang, Suyeon Seo, Eunsol Park, Min-Woo Han

**Affiliations:** Advanced Manufacturing & Soft Robotics Lab, Department of Mechanical Engineering, Dongguk University, 30 Pildong-ro 1, Seoul 04620, Republic of Korea

**Keywords:** soft robots, residual stress, buoyancy control, gripping robots, pneumatic actuation

## Abstract

Underwater soft robots offer many potential applications, including exploration, search, and rescue missions. Notably, these recently developed underwater soft robots present a safer and more adaptable alternative to rigid robots currently in use. Their flexible and deformable bodies enable them to easily adapt to challenging underwater environments and interact with diverse aquatic creatures and structures. In this paper, we present a soft buoyancy gripper that can manage buoyancy and adjust its position in the water without relying on external mechanisms. Modulating the volume of internal fluid can function both as a gripper and adjust buoyancy as needed. When buoyancy is reduced and fluid volume is minimized, the gripper can securely grasp objects, while increased fluid volume and buoyancy allow for delicate object placement. During experiments, the gripper successfully grasped and released multiple objects. When an extra channel was added, the crawling motion was achieved. The buoyancy control system demonstrates versatility and adaptability, offering the possibility of safe underwater exploration and research. Its ability to operate without harming marine environments or organisms makes it suitable for underwater research.

## 1. Introduction

Water hyacinth possesses distinct structural characteristics that enable it to float on water. The petiole is a spongy stem capable of absorbing air and supports the leaves and flowers above the water surface. The petiole has buoyancy due to its air-injectable channels, allowing the water hyacinth to remain afloat. The injected air enables the plant to rise to the surface of the water. Throughout the plant, including the stem, roots, and leaves, air-filled dermal tissues are distributed. This not only facilitates the plant’s ability to float on the water surface but also contributes to its overall buoyancy. These structural attributes enable the leaves of the water hyacinth to be positioned on the water surface, directly exposed to sunlight, thereby enabling photosynthesis [1,2]. The proposed robot in this paper mimics the functional integration of the water hyacinth’s petiole, where the internal air-filled tissues (aerenchyma) provide buoyancy. Our bilayer design replicates this by using the Dragon Skin layer for structural curling and the Ecoflex layer for volumetric expansion.

Conventional submarines and rigid robots use main ballast tanks made of metallic materials such as steel, adjusting their underwater position by regulating the ratio of seawater and compressed air. To replace this system, various soft robotic mechanisms have been proposed for underwater position control [3,4,5,6].

Soft robots, characterized by their softness and flexibility, are suitable for realizing efficient movements found in nature, and various studies are being conducted to implement these in underwater environments [7,8]. Soft robots inspired by aquatic organisms have unique properties to achieve underwater functionality [9,10,11,12]. Inoue et al. introduced an underwater robot that regulates buoyancy by leveraging the spermaceti oil found in sperm whales. This approach mimics the mechanism employed by sperm whales, which control their buoyancy by melting or solidifying the oil in the spermaceti organ located in their heads [13]. Swimming Leaf is an underwater robot that takes the shape of a leaf and swims through the water with a fluttering motion. Mimicking the buoyancy mechanism of cuttlefish determines buoyancy by adjusting the specific gravity of liquid and air through osmosis [14]. The robotic soft swim bladder is a soft buoyancy device that mimics the ability of bony fish to move up and down in water. When the heating element operates, the liquid and vapor inside the buoyancy pouch undergo a phase transition, changing the volume and density to control buoyancy [15].

Some underwater soft robots mimic propulsion mechanisms found in aquatic animals, using fluid dynamics for efficient movement. A spherical underwater robot inspired by squids moves with water jets for locomotion, enabling underwater position control through bidirectional water ejection from nozzles. It generates propulsion by retaining a large volume of water and subsequently expelling it, thereby achieving position changes in aquatic environments [16]. PoseiDRONE is a soft underwater robot that mimics the movement of an octopus. It simulates the leg movements of an octopus with four silicone legs and can crawl or swim underwater. Swimming is achieved through pulse jet propulsion, driven by the expansion and contraction of fluid within an elastic chamber. Each leg is capable of realizing crawling motion with a three-bar mechanism and moves by applying a force opposite to the ground at the end of the leg [17].

For the flexible movement of soft robots, materials with high elasticity and resilience are essential. The representative material is polymer, widely used to fabricate various soft robots [18,19,20,21]. Polymers, including Ecoflex and Dragon Skin, have been widely used in the fabrication of various soft robots [22,23,24]. They are lightweight, stretchable, and can be molded into desired shapes, allowing intentional generation of residual stress. Robots based on residual stress with pneumatic channels within polymers can change their shape through external forces [25]. Soft polymers can also be utilized in immediate actuation systems, such as pneumatic control [26]. Pneumatics, a representative actuation source in soft robotics, enables rapid and versatile shape deformation in flexible fluidic actuators [27]. These actuators typically consist of an elastomer with multiple embedded channels that deform under pneumatic pressure [28,29]. This approach has also been adopted in underwater grippers, which gently grasp objects utilizing pneumatics [30,31]. Unlike conventional soft grippers, our design reduced system complexity with a single active body.

This paper proposes a soft robot that can be used underwater, reflecting the structural characteristics of water hyacinths. By utilizing residual stress within a bilayer structure, the developed soft robot is capable of both vertical position adjustment and stable grasping control in water. This programmed morphology of a bilayer structure to integrate dual functions of depth and grasping control eliminates the need for heavy mechanical ballast tanks and shows a lightweight soft structure. Air-injectable channels are designed within the elastic polymer. When air is injected through the air tube connected to one side of the soft robot, buoyancy control in water becomes possible. It allows position adjustment from the water surface to the bottom. Additionally, bending and unfolding motions can be generated by the activation of air channels. It is capable of grasping objects by controlling the air channels, and a crawling motion is also available even on land. Since it has a soft and flexible polymer structure actuated by pneumatics, this soft robot can operate in both aquatic and terrestrial environments. It can interact with the external environment and perform various movements and tasks through channel diversification. Furthermore, it is expected to be compatible with devices and underwater robots that need to interact with various sea creatures.

## 2. Materials and Methods

**Figure 1:**
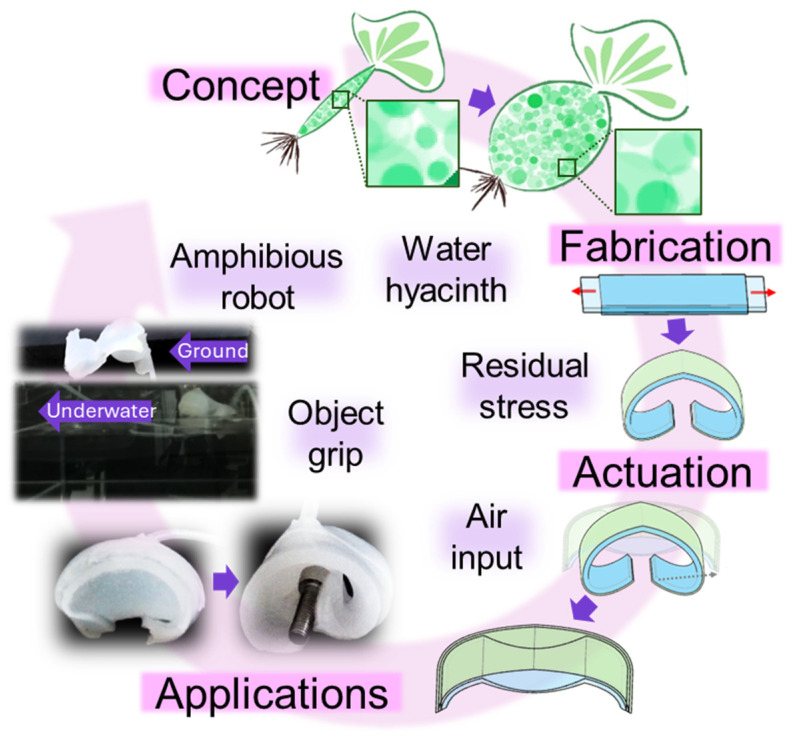
Rapidly expanding stress-driven soft robot based on air injection.

The soft robot designed in this study is composed of soft materials that allow its shape to change based on air injection (Figure 1), inspired by the structural characteristics of water hyacinth, specifically its buoyancy.

Soft robots constructed from compliant materials generally have unlimited degrees of freedom and can safely interact with objects. By applying pre-stretch to polymer materials, the robot gains residual stress, which induces a curling state. This fabrication allows the robot to grasp objects by alternating between relaxed and stressed states using air pressure.

Residual stress refers to the stress that remains inside a material even after an external force that deformed the material is removed. The more the structure is stretched, the stronger its tendency to return to its original state, generating more residual stress. This residual stress plays a major role in forming the curvature of the soft robot. When air is injected into the rolled soft robot, which retains residual stress, it swells, and the curvature decreases. The magnitude of residual stress directly influences the curvature and shape change of the robot.

As a method for generating residual stress, two different polymers were used in the fabrication process. The softer material was stretched and then fixed by curing a stiffer polymer on top so that the residual stress remained in the structure. The mechanical properties of the two polymers used in this study are shown in Table 1. Therefore, a thin membrane made of Ecoflex is stretched, and Dragon Skin is poured onto it. Residual stress is formed as Dragon Skin is cured on top without resolving this deformation. As a result, the size and shape of the soft robot can be controlled based on the amount of air injected, leading to changes in its position underwater. The flexibility of the polymer material allows the robot to have varying buoyancies depending on the volume of air injected. In addition to functioning underwater, the robot can be utilized as a soft gripper, capable of lifting and lowering objects both in and out of water.

**Figure 2:**
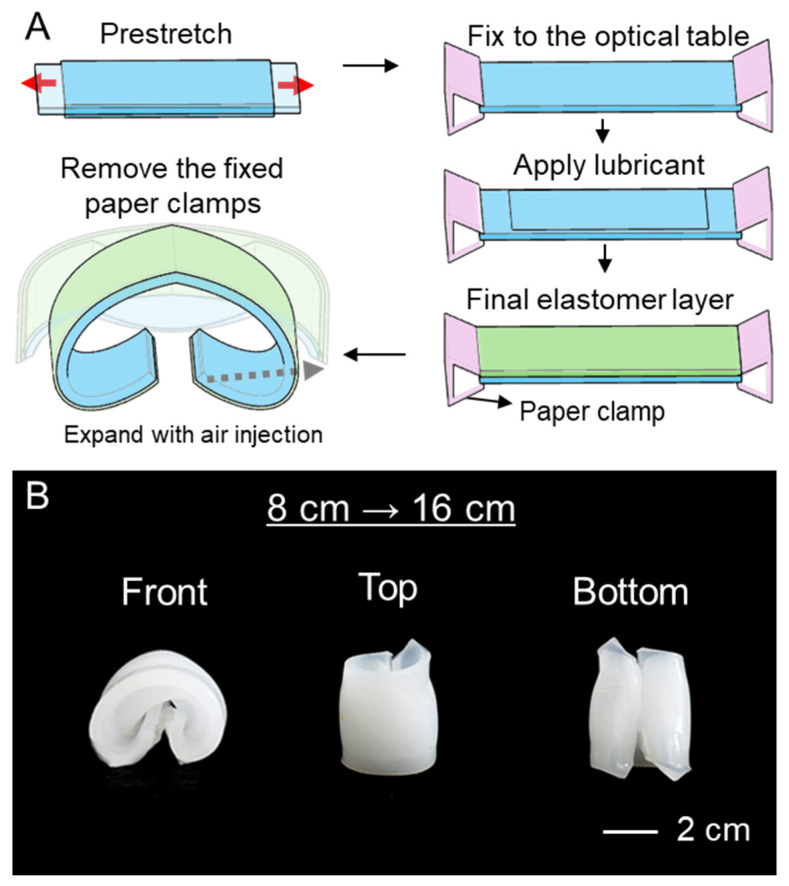
(**A**) Fabrication method of soft robot with residual stress. (**B**) Photographs of the soft robot after fabrication. Front, top, and bottom views are shown.

The polymer was stretched from its initial length, using paper clamps to fix it in place during curing (Figure 2A). This tensioned and fixed polymer membrane maintains residual stress due to the elastic restoring force during the curing process of the upper polymer layer [25]. After the clamps are removed, the soft robot maintains a curled shape due to the residual stress created by the difference in elasticity between the two polymer layers. When air is injected into the air layer between these layers, the robot expands into a linear structure. To model the residual-stress-induced curvature of the bilayer soft actuator, we approximate the strain. The initial structure consists of Ecoflex 30 with a width $W_{0}=8\;cm$, length $L_{0}=6\;cm$, and thickness $t_{s}=4\;mm$. This structure was stretched up to W=16 cm, and the strain generated during tension is as follows.

$$\varepsilon =\frac{W-W_{0}}{W_{0}}$$

Then, with both ends fixed, Dragon Skin with a thickness of $t_{f}=2\;mm$ was poured into the upper part and cured at room temperature. After curing, the fixing was released. At this point, the curvature formed due to the mismatch in the strain between the upper and lower layers. Egunov et al. presented the curvature κ formed in the PDMS-based bi-layer film by following the theoretical equation [34].

$$\kappa =\frac{6\delta }{H}\cdot F(n,m)$$

Here, δ=ε is a function determined by the mechanical strain by tension, $H=t_{s}+t_{f}=6\;mm$ is the overall thickness, and F(n,m) is the latter thickness ratio from $n=t_{f}/t_{s}$ and elastic modulus ratio $m=E_{f}/E_{s}$.

The radius of curvature was calculated assuming the initial membrane radius as $R_{0}=35\;mm$ and the pre-stretch ratio $\lambda_{0}=2.0$.

$$R_{cap}=\frac{\lambda_{0}R_{0}}{sin\theta }$$

The curvature is as follows:

$$k=\frac{1}{R_{cap}}$$

For the rectangular membrane model, in the case of a long rectangular membrane (length to width ratio > 5), the center displacement at uniform pressure *p* can be expressed by the following equation.

$$\frac{\delta }{b}={\frac{1}{8}\left[\frac{24\left(1-\mu^{2}\right)pb}{Et}\right]}^{1/3}$$

Here, b is the length, t is the membrane thickness, E is the modulus of elasticity, and μ is Poisson’s ratio. The radius of curvature R of the arc consisting of string b and height δ is derived by the following geometry.

$$\delta (vertical\;distance)=R-\sqrt{R^{2}-{(\frac{b}{2})}^{2}}$$

This enables the robot to not only grasp small objects but also function as a crawling robot capable of moving on water and land.

In experiments, a thin polymer layer with initial dimensions of 8 cm × 8 cm was stretched to lengths of 16 cm. The degree of curling was observed to increase with the amount of pre-stretch applied, as shown in Figure 2B. Figure S1 shows the relationship between the pre-stretch ratio and curvature. As the pre-stretch ratio increased from 1.25 to 2, the curvature increased linearly from 0.53 to 0.6. Curling is particularly advantageous when the robot is used for grasping small objects.

Buoyancy is controlled by adjusting the volume of the air layer formed between the first and second polymer layers. When air is injected into the curled soft robot underwater, the volume of the air layer increases, expanding the internal volume. It causes the robot to rise toward the water’s surface by generating buoyancy. In the absence of air, the robot sinks to the bottom, but as air is injected, it gradually ascends and can be positioned at different depths. Moreover, by injecting air while grasping an object, the robot can release the object and simultaneously rise to the surface. To assess the application value of the proposed soft robot, the energy consumption for buoyancy control was analyzed in Section S1.

## 3. Results

### 3.1. Deformation Experiments

**Figure 3:**
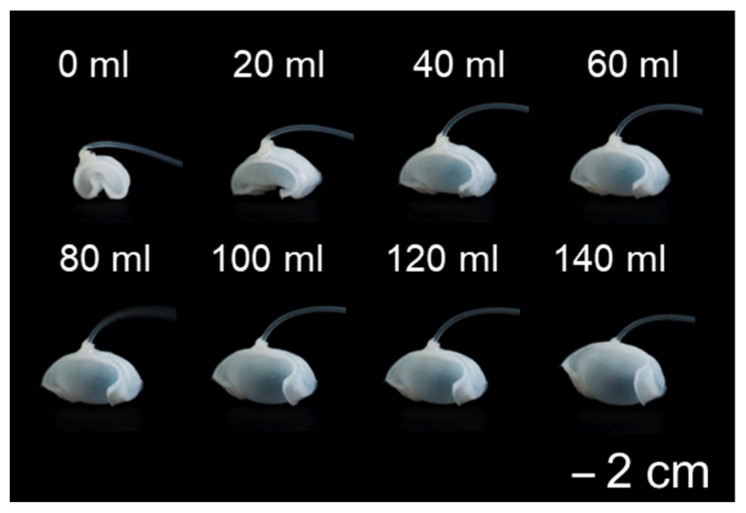
Deformation sequence of the soft robot induced by air injection.

The shape of the soft robot changes in response to air injection. Figure 3 shows the deformation behavior of the soft robot when the amount of air is increased by 20 mL.

In this experiment, a syringe was used to inject 140 mL of air into the specimens. The radius of curvature was measured to see the differences due to air injections (Figure S2). It shows a reduced curvature with higher air input.

### 3.2. Underwater Position Control with Air Injection

**Figure 4:**
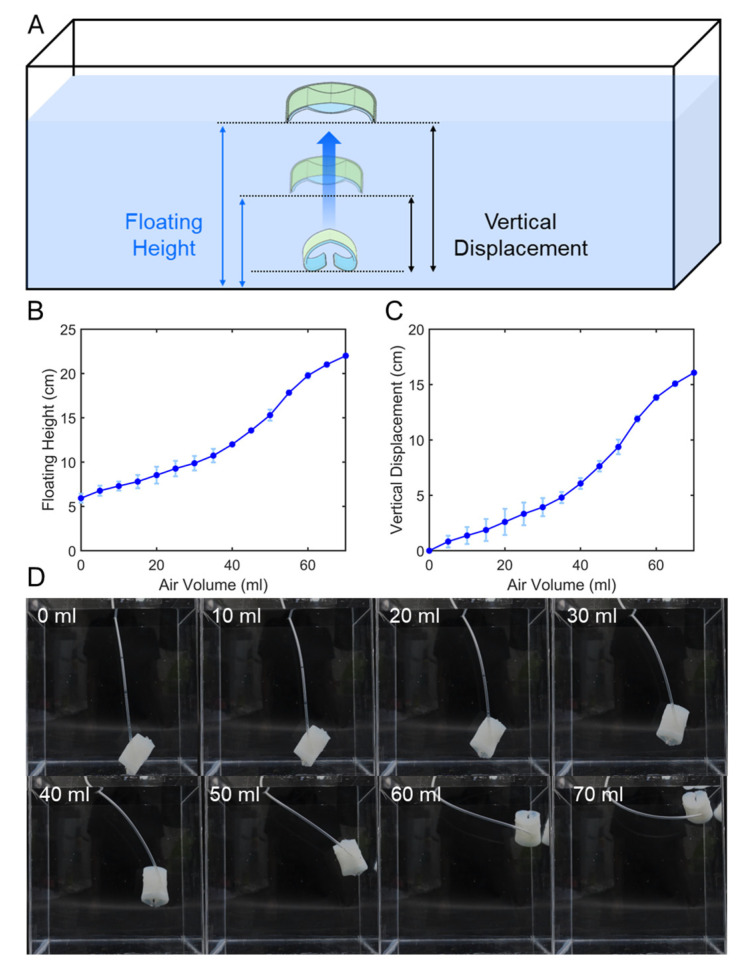
(**A**) The definition of floating height and vertical displacement is shown. (**B**) Underwater position when air is injected at a constant rate of 20 m. (**C**) Vertical displacement ($∆h=h-h_{initial}$) from graph (**B**). (**D**) Captured images of the underwater position with air injection.

The change in underwater position can also be observed as the area receiving buoyancy increases with the shape change when air pressure is applied (Figure 4A). The shape and position changes with 20 mL increments can be seen in Figure 4B,C. The position in the water rises proportionally as air is injected. Figure 4C highlights the changes in underwater position based on the floating height shown in Figure 4B. The standard deviation for the repeated experiments was approximately 0.55 cm. Figure 4D shows the captured images of the underwater position with air injection.

**Figure 5:**
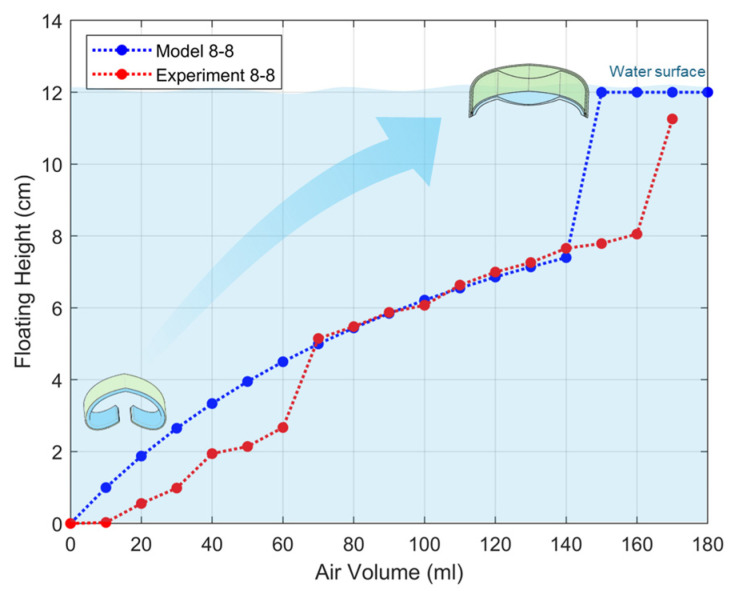
The calculated floating height in the water and experimental data.

Video S1 shows the repeated experiments of position change in water with varying air volumes for an 8–16 specimen (Ecoflex membrane stretched from 8 cm to 16 cm). This demonstrates that the device can be used repeatedly for buoyancy control. In addition, the floating height of the soft robot according to the air input was calculated and shown together with the experimental data as a graph (Figure 5). The underwater location of the soft robot was calculated using the buoyancy formula. Here, to simplify the calculation, a specimen was made without pre-stretch as shown in S3. S4 shows the specimen swells with air injection. The buoyancy of the soft robot follows Archimedes’ principle. Buoyancy is calculated using the volume of the object being submerged and the density of water.

$$F=\rho_{water}\cdot V\cdot g$$

Here, $\rho_{water}$ is the density of water, V is the volume of an object, and g is the gravitational acceleration. The height of the water tank was set to 21 cm, the same as in the experiment. The total volume of the soft robot increases as air is injected, and it is calculated as follows.

$$V_{total}=L\cdot W\cdot (t_{ecoflex}+t_{dragonskin})+V_{air}$$

Here, L is the length, *W* is the width of the soft robot, $t_{ecoflex}$ is the thickness of Ecoflex, $t_{dragonskin}$ is the thickness of Dragon Skin, and $V_{air}$ is the volume of air injected. The total mass of the object was calculated as follows:

$$m_{total}=m_{ecoflex}+m_{dragonskin}$$

Here, $m_{ecoflex}$ is the mass of Ecoflex and $m_{dragonskin}$ is the mass of Dragon Skin. The model results show a similar trend to the experimental data, but a sudden change is observed after 140 mL of air injection. This is because the volume of the object satisfies the buoyancy for floating in water at 140 mL.

### 3.3. Variation with Weight

**Figure 6:**
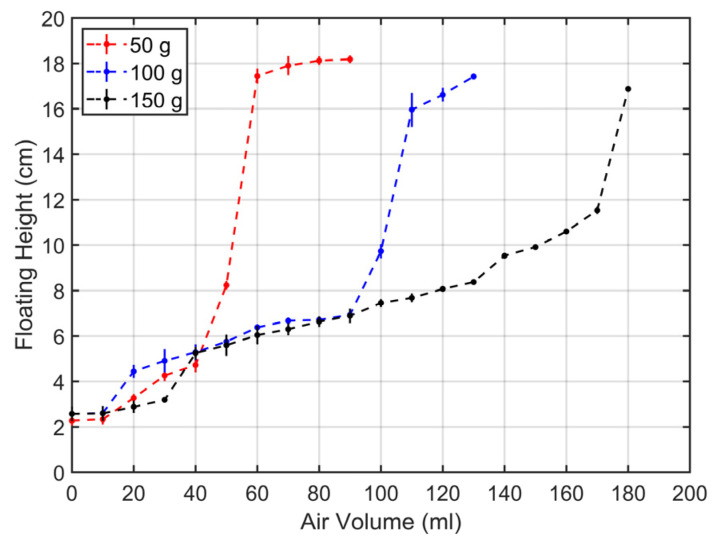
Underwater position with weights attached when air is injected at a constant rate of 20 mL.

Experiments were conducted on each specimen with weights of 50 g, 100 g, and 150 g to observe the position change in water according to the amount of air. The results in Figure 6 showed that using a 50 g weight allowed for rapid upward movement with a small amount of air. Additionally, it was determined that heavier weights need more time to control the position in the middle of the underwater. As the stress increased, the surface area decreased, leading to a rapid change in water position with the amount of air.

### 3.4. Gripping Actuation

**Figure 7:**
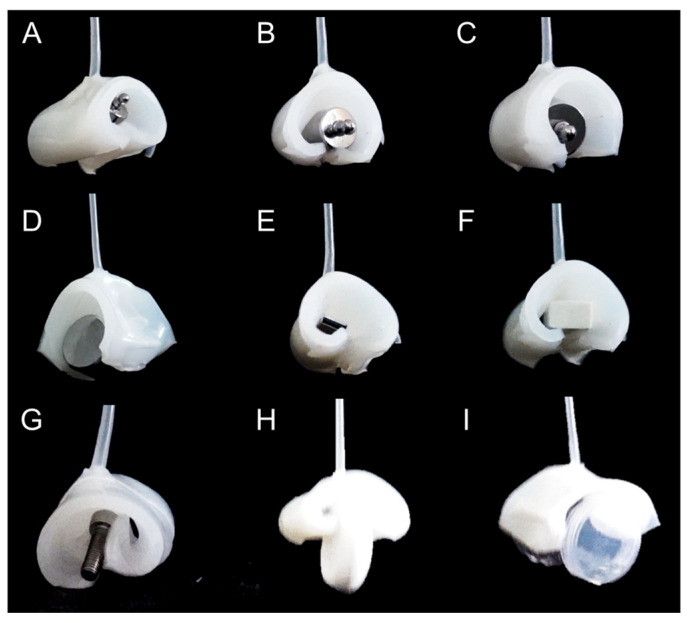
Gripping actuation of the soft robot with various lifting objects (**A**) 20 g Weight; (**B**) 50 g Weight; (**C**) 100 g Weight; (**D**) 200 g Weight; (**E**) USB; (**F**) Eraser; (**G**) Screw; (**H**) Floss Case; and (**I**). Cylinder.

Next, the gripping actuation for lifting objects was examined. Experiments were conducted to lift common objects as shown in Figure 7. These included learning supplies such as erasers and USB drives, as well as engineering items like weights and screws. The objects ranged in size from approximately 1 cm to 5 cm and in weight from 4.5 g to 200 g (Table 2). The soft robot maintained its rolled shape without applying air pressure, allowing it to hold small objects without dropping them. To grip an object, the robot descends to the object’s position and applies air pressure to change to a straightened state. Then, it descends slightly further into the straightened state, removes the air pressure, and changes back to a rolled state, securely holding the object inside. Afterward, it can lift and move the object. To release the object, the robot descends to the desired position and changes to a straightened state by applying air pressure. Objects can also be released underwater, which can be useful for moving objects from outside to inside the water. It is also possible to drop objects while gripping them underwater, which can be used to indicate the location where the objects were dropped on the water surface. The actuator was found to be effective when lifting small but heavy objects like screws and weights. Video S2 shows a detailed demonstration of lifting and lowering the smallest object, a screw. Figure 7 also shows the stable lifting and lowering of a screw and dental floss. The image shows that a cup can be lifted and landed stably.

### 3.5. Application as an Amphibious Robot with the Addition of Channels

**Figure 8:**
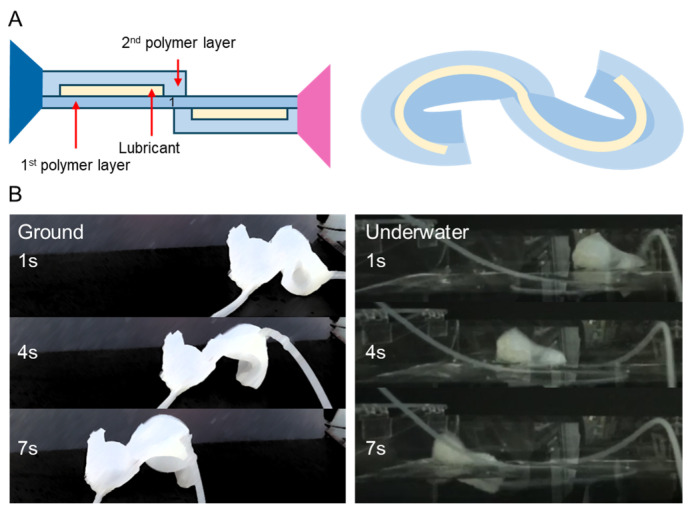
(**A**) Structure of residual stress robot. (**B**) Movement of an amphibious robot on the ground and in water.

By adding one more channel to this robot (Figure 8A), it can be applied as an amphibious soft robot with two channels. As air is injected into the two channels alternately, the soft robot’s front and back parts expand and contract, enabling forward movement (Figure 8B). This movement can be controlled by the structural flexibility of the soft robot and the pneumatic input, and it can operate on both land and water. Video S3 shows an amphibious soft robot walking into the water. The amphibious soft robot can move flexibly and efficiently even in water, performing various tasks, and it can be utilized in various applications such as marine exploration, underwater structure inspection, and environmental monitoring.

## 4. Discussion

The residual stress utilized in this work proposed a simple design approach for programming complex morphologies in soft structures. While the current study focuses on using curvature for grasping and buoyancy control, similar methods have been explored in prior work to achieve shape-morphing capabilities using bilayer bending and pre-strained elastomers [35,36]. However, most of these approaches have been limited to a single functionality.

This study demonstrates the integration of two distinct functions into a single soft body, which are grasping and buoyancy control. It is enabled by residual stress and pneumatic actuation. In contrast to previous designs, where object manipulation and buoyancy control are handled through separate components or systems [37,38], the presented robot uses a unified structural design, simplifying fabrication and control. This reduced hardware complexity in soft robotics while enhancing multifunctionality.

In this study, we developed an underwater soft robot with residual stress. It is controlled by simple air injection, changing between a stressed and relaxed state. Fabricated by pre-stretching a polymer membrane and curing a second layer on top, it can both function as a soft gripper and as a buoyancy control device. The design of the robot created a stable, curled structure using residual stress. The degree of curling can be adjusted by changing the length of pre-stretching. By adding pneumatic channels, the robot shows locomotion capabilities even on the ground and in water. The robot safely interacts with its entirely soft materials, making it ideal for applications in aquatic environments. This work demonstrates an approach to designing soft robots that are capable of complex motion through simple fabrication and control. The proposed design can be further optimized for underwater location control to improve performance in underwater exploration and manipulation tasks.

However, there are some limitations, such as the lack of real-time feedback control for precise positioning and the potential degradation of mechanical properties over extended operational cycles. While we confirmed the consistent performance of the robot over 50 operational cycles using an integrated flex sensor (Figure S5), long-term degradation of mechanical properties under much higher cycle counts remains to be explored. Additionally, the current experiments were conducted in static water; the performance in complex environments such as flowing water and turbid water remains to be explored. Also, the current study remains at a conceptual stage; the integration of an additional pneumatic channel suggests a potential for multi-modal locomotion, such as crawling. Detailed kinematic analysis and systematic experimental validation will be pursued as future work.

## Figures and Tables

**Table 1:** Material properties of Ecoflex and Dragon Skin [32,33].

Properties	Ecoflex 30	Dragon Skin 30
Specific Gravity	1.07 g/cc	1.08 g/cc
Tensile Strength	200 psi	500 psi
100% Modulus	10 psi	86 psi
Shrinkage	<0.01 in./in.	<0.01 in./in.
Shore hardness	30A	30A
Color	Translucent	Translucent
Pot life	45 min	45 min
Cure time	240 min	960 min
Manufacturer	Smooth-On	Smooth-On

**Table 2:** Target objects included learning supplies and engineering items.

	Target Objects	Diameter × Height (mm × mm)	Weight (g)	Shape Regularity
A	20g Weight	15 × 23	20	Slightly irregular
B	50g Weight	20 × 31	50	Slightly irregular
C	100g Weight	25 × 38	100	Slightly irregular
D	200g Weight	35 × 48	200	Slightly irregular
E	USB	12 × 42 × 5	4.50	Highly irregular
F	Eraser	18 × 39 × 10	10.96	Highly irregular
G	Screw	10 × 20	6.13	Highly irregular
H	Floss Case	50 × 18	12.05	Slightly irregular
I	Cylinder	48 × 70	16.73	Regular

## Data Availability

The original contributions presented in this study are included in the article/Supplementary Material. Further inquiries can be directed to the corresponding author.

## Supplementary Materials

The following supporting information can be downloaded at: https://www.mdpi.com/article/10.3390/biomimetics11050317/s1. Video S1. Soft robot that moves up and down underwater. Video S2. Soft robot that lifts and puts down the screw. Video S3. An amphibious soft robot that moves between water and the floor. Figure S1. Curvature of the soft robot as a function of pre-stretch extension. Figure S2. The radius of curvature decreases with air input. Figure S3. Fabricated specimen without pre-stretch. Figure S4. Specimen from S2 with air injection. Figure S5. The resistance values from the integrated flex sensor demonstrate consistent performance over 16 (top) and 57 (bottom) operational cycles. Raw sensor data were processed with drift compensation to account for the inherent characteristics of the flex sensor. Table S1. Performance comparison between the proposed soft robot and existing underwater soft robot. Section S1. Energy consumption analysis.
