# Supplementary material for: Residual Stress-Based Soft Robot with Capability for Grasping and Buoyancy Control
Source: Biomimetics (Basel). 2026 May 2;11(5):317. doi: 10.3390/biomimetics11050317 (PMC13204773; doi:10.3390/biomimetics11050317)
Supplement: Supplementary file 1 [file biomimetics-11-00317-s001.zip › biomimetics-4190675-supplementary-english.pdf]

Residual Stress-Based Soft Robot with Capability for Grasping and Buoyancy Control

Minchae Kang[1], Suyeon Seo[1], Eunsol Park[1] and Min-Woo Han[1,*]

[1] Department of Mechanical Engineering, Dongguk University, Seoul, Republic of Korea

*Author to whom any correspondence should be addressed.

E-mail: mwhan@dgu.edu

Supplementary information

Video A. Soft robot that moves up and down underwater

Video B. Soft robot that lifts and puts down the screw

Video C. An amphibious soft robot that moves between water and the floor

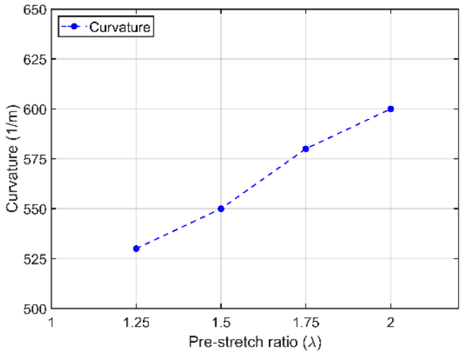

Figure S1. Curvature of the soft robot as a function of pre-stretch extension.

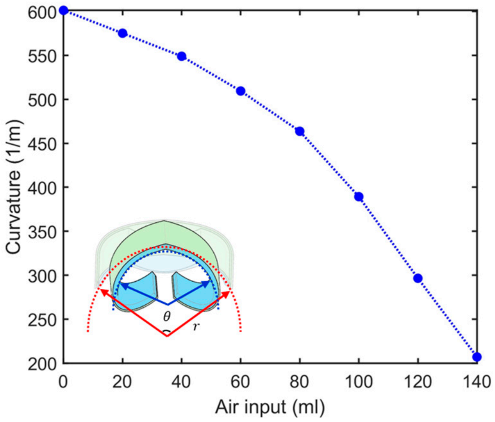

Figure S2. The radius of curvature decreases with air input.

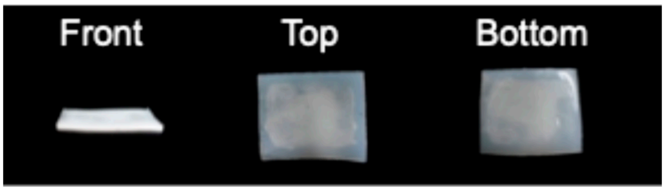

Figure S3. Fabricated specimen without pre-stretch.

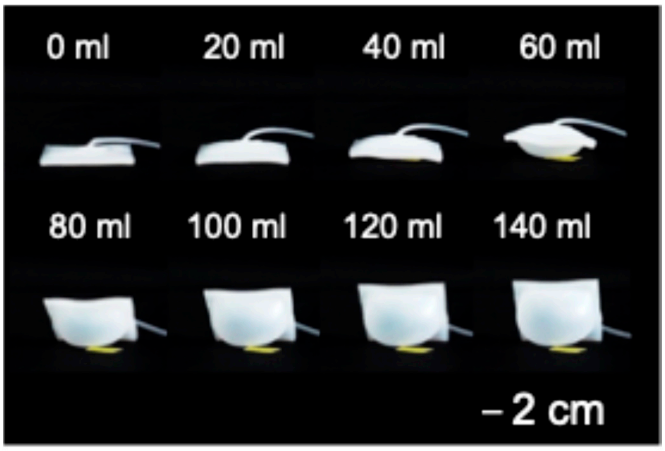

Figure S4. Specimen from S2 with air injection.

Figure S5. The resistance values from the integrated flex sensor demonstrate consistent performance over 16 (top) and 57 (bottom) operational cycles. Raw sensor data were processed with drift compensation to account for the inherent characteristics of the flex sensor.

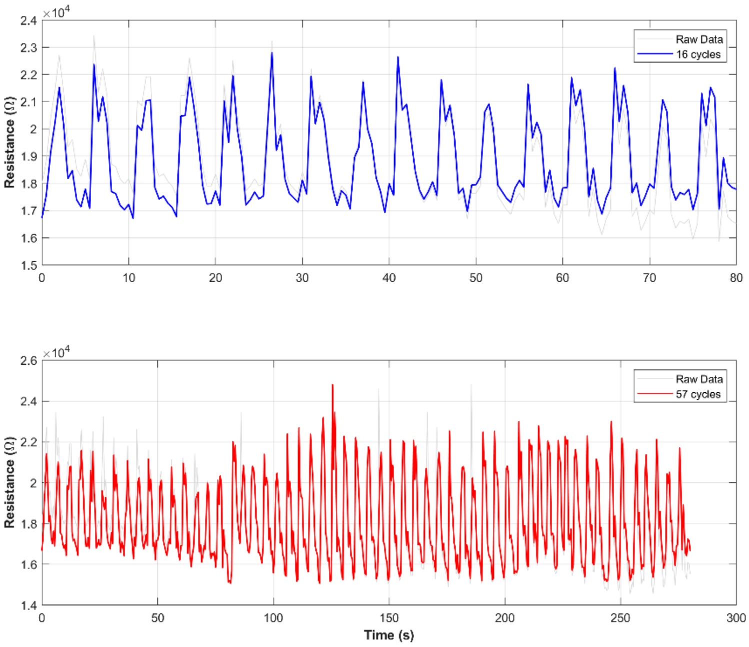

Table S1. Performance comparison between the proposed soft robot and existing underwater soft robot

Comparison Criteria	Proposed soft robot	Wu et al.	Sinatra et al.
Actuation Mechanism	Bending driven by residual stress between two polymer layers	Pump driven soft suction disk actuated by 3D-printed linkages and tubular bellows	Nanofiber reinforced silicone matrix driven by hydraulic pressure
Hardware Complexity	Low. Fabricated simply by stretching a soft polymer and curing a stiffer polymer on top	High. Requires modular mold casting, a complex 3D-printed linkage mechanism	Moderate. Requires nanofiber spinning, sequential co bonding of silicone layers and attachment to a 3d printed palm hub
Manipulation	Capable of lifting various objects, including screw and safe due to soft materials but relies on elastic restoring stress which lacks fine tuned low pressure control for extreme fragility	Capable of nondestructive capture of fish and turtles, but specifically engineered to stay below 1kPa of contact pressure	Specifically design for gelatinous marine organisms, maintaining an ultra-low contact pressure of ~0.0455 kPa
Material	Silicone polymer Ecoflex 30 Dragon skin 30	Silicone (suction disc), TPU (tubular bellows), photosensitive resin (3D printed parts)	Silicone (Dragon Skin 20), polymer nanofibers (Nylon-6 polyurethane), and photopolymer (3D printed palm)
Presence of rigid components	None.	Yes. 3D printed linkage mechanisms, a slider rod, and rigid stents inside the suction cups	Yes. 3D printed central hub (palm), stainless steel fasteners

Section S1. Energy consumption analysis

To evaluate the practical application of the soft robot, the energy consumption required for a buoyancy control cycle was estimated. The mechanical work required to expand the internal air chamber is defined by the integral of the net pressure over the volume change.

The work required to expand the internal air layer is

$$\boldsymbol{W} = \int_{\boldsymbol{V_i}}^{\boldsymbol{V_f}} \boldsymbol{P_{net}(v) dV}$$

where $\boldsymbol{P_{net}(v)}$ is the internal pressure required to overcome both the hydrostatic pressure and elastic restoring force of the bilayer membrane. $\boldsymbol{V_i}$ and $\boldsymbol{V_f}$ are the initial and final volumes, respectively.

For a maximum inflation of 140 mL at an average pressure of 3 kPa and a depth of 21 cm, the total energy consumed per cycle is approximately 0.43 J. This low energy requirement suggests that the robot can operate for extended periods using small-scale portable power sources. For instance, a compact 1.85 Wh battery could theoretically support over 15,000 inflation/deflation cycles, making it suitable for persistent environmental monitoring and search-and-rescue missions in confined aquatic spaces.
